# Important Item—Medical Education

**Published:** 1876-05

**Authors:** 


*Important Item—Medical Education*—We are glad to see that the RECORD and other journals are calling attention to the "manufacture" of an undue number of imperfectly educated medical graduates. The charge "that the colleges are the worst enemies of the profession," we fear could be fully sustained by the facts which are often stated in some of our journals, and unless a great reaction takes place, and that very soon, which will insure protection to the educated members of the profession, and citizens, a demand for government interference will become absolutely necessary. T. W. H.

The above remarks are from one of our oldest and most substantial friends. It is true as he states that we have protested against the "manufacturing of imperfectly educated medical graduates," and that the legitimate journals of the country everywhere are crying out against the rapid and alarming growth of this evil.

It seems, however, that all that has been said to remedy this evil amounts to but little. So long as the claims of superiority in the respective colleges are based upon the size of the classes and the number of graduates they can annually turn out, so long will the rivalry and competition in the matter of numbers lead to the graduation of imperfectly educated students. We have known medical professors to acknowledge, with regret, the anomalous and mortifying situation which the present system imposes—it being scarcely possible that a school can be successfully maintained which insists upon rigid examinations, and upon the elevation to a proper standard of the requirements necessary to fit the graduate for the responsible duties of a practitioner of medicine.

If fitness and merit of the graduates, and not numbers, could be made the standard of respectability for the colleges, there would be hope of a change for the better; and this can be done if the profession throughout the land, and the medical associations, would co-operate in the movement.

A remedy suggested is the transfer, by legal enactment, in the several States of the power of examining the applicants for graduation to independent Boards of examination. These Boards, having no connection with the colleges, and being under no inducement to make a show of numbers, would be much more likely to act impartially and justly, whilst the great effort of the colleges would then be to prepare their students for a good and successful examination before these Boards, and thus a true and proper spirit of rivalry would be encouraged, and merit and not numbers would become the controling element in these institutions. The schools would thus be stimulated to efforts in the right direction. The independent examining Boards would become the mediums through which the merits of the respective schools could be known, and students would no longer be misled by the show of numbers, and by high sounding advertisements of wonderful and superior facilities which do not exist. The legitimate and respectable colleges would be known to these Boards by their merits, and we could know through these Boards the true character of competing institutions. The students themselves would be stimulated to seek the highest attainments, and be enabled to learn where to seek them, while the old and faithful members of the profession throughout the land would be protected from the locust-like swarms of quacks and imposters that are annually poured out upon the country.

Now, we would not be understood as attaching all the blame to the colleges for this state of things. The profession and the societies, and the amazing ignorance and indifference of the masses in regard to medical science, as evinced in the imperfect legislation which is had upon this subject of licensing medical practitioners, all combine to bring about the unfortunate condi tion of things.

Many of our colleges no doubt desire to remedy this evil, and some of them have made, and are now making, efforts in this direction, but not being backed up by the profession and the law-making authorities, have failed to accomplish the desired object. If all the legitimate medical journals will advocate the establishment of independent medical Boards throughout the States as the first step toward the needed reform; and if the American Medical Association, in this Centennial year, will lead off in this, or some other measure which will fulfill the same object, and the medical societies throughout the land will follow in the wake, the result will be that a great and decided step will have been taken in the right direction, and we shall soon witness great and important advancement in the matter of medical education in this country.

